# Special Issue of the Manufacturing Engineering Society 2020 (SIMES-2020)

**DOI:** 10.3390/ma14123208

**Published:** 2021-06-10

**Authors:** Eva María Rubio, Ana María Camacho

**Affiliations:** Department of Manufacturing Engineering, Industrial Engineering School, Universidad Nacional de Educación a Distancia (UNED), St/Juan del Rosal 12, E28040 Madrid, Spain; amcamacho@ind.uned

**Keywords:** additive manufacturing, forming, joining, machining, green manufacturing, robotics, metrology, Industry 4.0, simulation

## Abstract

The Special Issue of the Manufacturing Engineering Society 2020 (SIMES-2020) has been launched as a joint issue of the journals “*Materials*” and “*Applied* *Sciences*”. The 17 contributions published in this Special Issue of Materials present cutting-edge advances in the field of Manufacturing Engineering, focusing on additive manufacturing and 3D printing; advances and innovations in manufacturing processes; sustainable and green manufacturing; manufacturing of new materials; manufacturing systems: machines, equipment and tooling; robotics, mechatronics and manufacturing automation; metrology and quality in manufacturing; Industry 4.0; design, modeling and simulation in manufacturing engineering. Among them, this issue highlights that the topic “advances and innovations in manufacturing processes” has collected a large number of contributions, followed by additive manufacturing and 3D printing; sustainable and green manufacturing; metrology and quality in manufacturing.

After the complete success of the first [1] and second editions [2,3] of the Special Issues of the Manufacturing Engineering Society (SIMES), with 48 and 39 (29 in *Materials* and 10 in *Applied Sciences*) contributions, respectively, on emerging methods and technologies, the Special Issue of the Manufacturing Engineering Society 2020 (SIMES-2020) [4] was relaunched as a joint issue of the same journals “*Materials*” and “*Applied Sciences*”.

Once again, this Special Issue was promoted by the Manufacturing Engineering Society (MES) of Spain [5], with the aim of covering the wide range of research lines developed by the members and collaborators of the MES and other researchers within the field of Manufacturing Engineering.

In this third edition, the joint issue has gathered a total of 31 papers in the topics presented in Figure 1, where the percentage of contributions of each topic to the Special Issue of the Manufacturing Engineering Society 2020 (SIMES-2020) is also shown.

Regarding the specific contributions of the Special Issue in the journal *Materials*, 17 contributions about cutting-edge advances in different fields of the manufacturing engineering have been collected. In particular, these concern advances and innovations in manufacturing processes [6,7,8,9,10,11]; additive manufacturing and 3D printing [12,13]; sustainable and green manufacturing [14,15]; metrology and quality in manufacturing [16,17]; manufacturing of new materials [18]; manufacturing systems: machines, equipment and tooling [19]; robotics, mechatronics and manufacturing automation [20]; Industry 4.0 [21]; design, modeling and simulation in manufacturing engineering [22]. Figure 2 shows the main topics and their percentages in this journal. 

Among all of them, the topic “Advances and innovations in manufacturing processes” stands out for the number of contributions it has had in this Special Issue (representing 35% of all of them), followed by the topics “Additive manufacturing and 3D printing”, “Sustainable and green manufacturing” and “Metrology and quality in manufacturing” (with a 12% each). The rest of the topics represent the remaining 30% of the contributions.

Concretely, within the topic “Advances and innovations in manufacturing processes”, the works are focused on manufacturing processes. Mainly, machining [6,7,8], forming [9], joining [10] and electroplating [11]. In machining, Kubo et al. [6] use bio-inspired DNA-based computing for determining surface topography of a dressed grinding wheel; Wood et al. [7] analyze the machinability of inconel718 alloy with a porous microstructure produced by laser melting powder bed fusion at higher energy densities; and Navarro-Mas et al. [8] compare different parameters to evaluate the delamination produces in the edge trimming of basalt fiber reinforced plastics. In forming, Merayo et al. [9], in order to characterize the plastic behavior of aluminum alloys, predict the mechanical properties of the material by artificial neural networks. In joining, Alves et al. [10] study attachment of sheets to tube ends made from dissimilar materials with a single stroke. Additionally, in electroplating, Tao et al. [11] analyze the effect of copper sulfate and sulfuric acid on the blind hole filling of high-density interconnect (HDI) circuit boards by electroplating.

Regarding the rest of the topics collected in the Special Issue, most are focused on “Additive manufacturing and 3D printing”. Alvarez et al. [12] study the direct generation of high-aspect-ratio structures of AISI 316L by laser-assisted powder deposition and Minguella-Canela et al. [13] study the manufacturing redesign of cooling inserts for high production steel molds and benchmarking with other industrial additive manufacturing strategies. Concerning “Sustainable and green manufacturing”, Colomer-Romero et al. [14] compare the mechanical properties of hemp-fiber biocomposites fabricated with biobased and regular composites, and Yagüe et al. [15] present a work on sustainable ecocements based on chemical and morphological analysis of granite sawdust waste as pozzolan. Concerning “Metrology and quality in manufacturing”, Mínguez-Martínez et al. [16] present a paper about design of industrial standards for the calibration of optical microscopes and Jiménez-Pacheco et al. [17] present research into the assessment of a gradient-based algorithm for surface determination in multi-material gap measurements by X-ray computed tomography.

The Special Issue collects five other works: in “Manufacturing of new materials”, Chaudhari et al. [18] analyze the effect of WEDM process parameters on the surface morphology of nitinol shape memory alloy; in “Manufacturing systems: machines, equipment and tooling”, Olmo and Domingo [19] show EMG characterization and processing in production engineering; in “Robotics, mechatronics and manufacturing automation”, Úbeda et al. [20] study the behavior of the force control loop used in a collaborative robot for sanding materials [20]; in “Industry 4.0” Ramirez-Peña et al. [21] make a descriptive review about the sustainability in the aerospace, naval, and automotive supply chain 4.0; in “Design, modeling and simulation in manufacturing engineering”, Curto-Cárdenas et al. [22] present a paper about the cold expansion process with multiple balls—numerical simulation and comparison with single ball and tapered mandrels.

Finally, it only remains to note that, in just five months since the publication of the first work [13], all the papers present a prominent activity in their “article metrics”. It is remarkable that some of the papers in this Special Issue have already more than a thousand views of the full-text and some of them even have citations, which is a clear evidence of the interest of all these topics in readers of the journal *Materials*, in general, and scientists and professionals from the industry in particular.

## Figures and Tables

**Figure 1:**
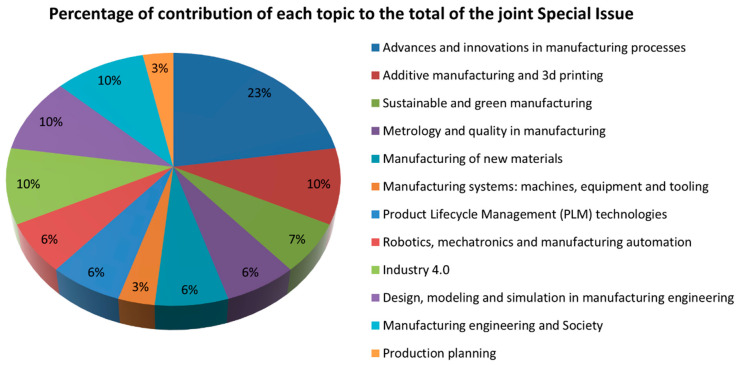
Percentage of contributions of each topic to the total of the joint Special Issue.

**Figure 2:**
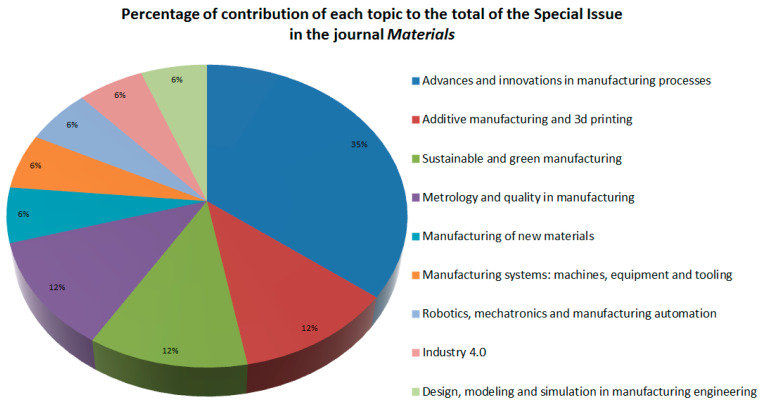
Percentage of contributions of each topic to the Special Issue in the journal *Materials*.
